# Risk of Tuberculosis Reactivation in Patients with Rheumatoid Arthritis, Ankylosing Spondylitis, and Psoriatic Arthritis Receiving Non-Anti-TNF-Targeted Biologics

**DOI:** 10.1155/2017/8909834

**Published:** 2017-06-01

**Authors:** Fabrizio Cantini, Carlotta Nannini, Laura Niccoli, Linda Petrone, Giuseppe Ippolito, Delia Goletti

**Affiliations:** ^1^Department of Rheumatology, Hospital of Prato, Prato, Italy; ^2^Translational Research Unit, Department of Epidemiology and Preclinical Research, National Institute for Infectious Diseases “L. Spallanzani”, Rome, Italy; ^3^Scientific Direction, National Institute for Infectious Diseases “L. Spallanzani”, Rome, Italy

## Abstract

Tuberculosis (TB) still represents an important issue for public health in underdeveloped countries, but the use of antitumor necrosis factor agents (anti-TNF) for the treatment of inflammatory rheumatic disorders has reopened the problem also in countries with low TB incidence, due to the increased risk of TB reactivation in subjects with latent tuberculosis infection (LTBI). Over the last 5 years, several non-anti-TNF-targeted biologics have been licensed for the treatment of rheumatoid arthritis, ankylosing spondylitis, and psoriatic arthritis. We reviewed the epidemiology of TB, the role of different cytokines and of the immune system cells involved in the immune response against TB infection, the methods to detect LTBI, and the risk of TB reactivation in patients exposed to non-anti-TNF-targeted biologics. Given the limited role exerted by the cytokines different from TNF, as expected, data from controlled trials, national registries of biologics, and postmarketing surveillance show that the risk of TB reactivation in patients receiving non-anti-TNF-targeted biologics is negligible, hence raising the question whether the screening procedures for LTBI would be necessary.

## 1. Introduction

Antitumor necrosis factor-targeted agents (anti-TNFs) infliximab and etanercept were licensed around 20 years ago, and over the following years, other anti-TNFs such as adalimumab, golimumab, and certolizumab pegol were approved. These drugs have changed the natural history of inflammatory rheumatic disorders including rheumatoid arthritis (RA), ankylosing spondylitis (AS), and psoriatic arthritis (PsA), with good control of symptoms and arrest or lowering of the disease progression. Nevertheless, it has long been recognized that the anti-TNFs are associated with increased risk of reactivation of latent tuberculosis infection (LTBI) [1–4].

In recent years, non-anti-TNF-targeted biologics, including anti-interleukin- (IL-) 1 anakinra (ANK), IL-6 inhibitor tocilizumab (TCZ), anti-CD20 rituximab (RTX), anti-CD28 abatacept (ABA), anti-IL-12 and IL-23 (UTK), and anti IL-17 secukinumab (SCK), were licensed for the treatment of RA (ANK, TCZ, RTX, and ABA), AS (UTK), and PsA (UTK and SCK). Since the use of ANK in RA is actually very limited due to its lower efficacy as compared with that of other biologics, data on TB risk associated with this biologic, previously discussed elsewhere [5], were not included in this manuscript.

The aim of this paper was to assess the tuberculosis (TB) risk in patients with rheumatic diseases receiving non-anti-TNF-targeted biologics. Moreover, the epidemiology of TB and the role of different cytokines (TNF-*α*, IL-6, IL-17, IL-12, and IL-23) in immune response against Mycobacterium tuberculosis (MTb) were reviewed.

## 2. Methods

A systematic review of the literature using PubMed database was performed to identify English-language articles related to all clinical trials and data from postmarketing surveillance and from national registries of currently employed non-anti-TNF-targeted biologics for rheumatic diseases to identify all cases of TB complicating the underlying rheumatic disease course. Data were extracted from phase II- and III-randomized controlled trials of at least a 12-week duration, their extension phase studies, and from prospective, open-label studies focused on the efficacy and safety of each drug. In addition, available data from biologic national registries, national healthcare databases, and postmarketing surveillance surveys were included. Reviews and meta-analyses were excluded. In the absence of data from registries and postmarketing surveillance, single-case reports of TB occurrence during the treatment with the most recent biologics were included. The following drugs were investigated: TCZ, RTX, ABA, UTK, and SCK. The research was performed by crossing the single drug with the following key terms: epidemiology, TNF-*α*, IL-6, IL-17, IL-12, IL-23, latent tuberculosis infection, tuberculosis, infections, comorbidities, and safety. The number of publications, the type of trial, the number of enrolled patients, the number of TB cases, and, when possible, the setting where TB cases occurred were recorded for each biologic. Moreover, the most relevant literature on TB epidemiology, immune mechanisms against TB infection, and methods to detect LTBI was reviewed. The literature review was extended to December 31, 2016.

## 3. Results

### 3.1. Epidemiology of TB in Different Countries

TB is still a leading cause of morbidity and mortality in the world [6], accounting for about 10.4 million new cases and 1.4 million deaths annually. Of note, more than two thirds of the global TB burden is reported in Africa and Asia, and in absolute terms, six countries accounted for 60% of the new cases: India, Indonesia, China, Nigeria, Pakistan, and South Africa. The poorest and socially excluded groups own the largest burden of disease emphasizing the need to invest on the management of the social determinants of health through poverty reduction measures and targeted interventions on high-risk populations.

The spread of multidrug-resistance TB requires special attention [7] and highlights the need to foster research on TB diagnostics, new drugs, and vaccines [8]. Although many advances have been made in the fight against TB over the last twenty years, a lot is still needed to achieve global elimination [8–11].

### 3.2. Role of Different Cytokines (TNF-*α*, IL-1, IL-6, IL-17, IL-12, and IL-23) in Immune Response against MTb

Although many components of the host immune response against MTb are known, the specific biomarkers and mechanisms underlying protective immunity remain obscure [8, 12, 13]. In addition to host and environmental factors, the genetic variation in MTb also plays a role in the clinical phenotypes of TB [14]. However, little is known about the interaction between human and MTb genetic diversity, and it has been argued that new paradigms and new conceptual frameworks are required to better understand and ultimately better control TB globally [15].

Upon MTb infection, active TB develops in some patients, whereas others contain the initial infection, and the disease is considered latent (asymptomatic). Among those latently infected, 5 to 10% will progress to active TB. Current understanding suggests that in subject with LTBI, which are estimated to be one fourth of the world's population [16], the infection is controlled by an active host immune system, whereas in patients with active TB, there is an uncontrolled bacterial growth due to an ineffective immune response which relies on the cooperation between innate and adaptive immunity. Until recently, LTBI was thought to represent a uniform state. However, it has become clear that LTBI has to be considered as a broad spectrum of infection states that differ by the degree of the pathogen replication, host resistance, and inflammation [17–19]. Although the immune system controls the infection, this control does not necessarily lead to sterilization. Once MTb is in the macrophages, the protective immune response against mycobacteria is dependent on the interaction between these host cells and CD4^+^ T cells. T cell-mediated immune response begins after the dissemination of MTb to the lymph nodes [20, 21]. Here, the antigen-specific T cells proliferate and then migrate to the infected lungs where they are found, together with other leukocytes, as part of the granulomas. Several distinct types of T helper (Th) cells (Th1, Th2, Th17, and regulatory T cells) are present at the site of infection.

The main Th1 cytokines are interferon-*γ* (IFN-*γ*), IL-12, and TNF-*α*. IFN-*γ* is mainly produced by the CD4 T cells whereas IL-12 and TNF-*α* by the antigen presenting cells (APCs). Th1 cells play an essential role in MTb control through the IFN-*γ* secretion enhancing the macrophage microbicidal mechanisms because they activate signaling pathways that include the inducible nitric oxide synthase (iNOS) pathway [22] and induce the process of acidification and maturation of phagosomes and autophagy [23–26]. IFN-*γ* is crucial for the defense against MTb. Individuals with mutations in the IL-12/IFN-*γ* axis develop disseminated infection caused by BCG or nontuberculous species of mycobacteria [27].

Th17 cells are characterized by production of IL-17A/F and IL-22, have strong proinflammatory capacities, and play a significant role in mucosal immunity. In animal models of TB, the presence of Th17 cells was associated with protection, and removal of IL-17-producing cells enhanced recruitment of Th1 cells to the lung [28]. IL-17 has been shown to have a protective immunity against hypervirulent MTb strains [29]. In addition, the magnitude of the Th17 response was found to be important, since mice repeatedly exposed to MTb and BCG developed strong IL-23-induced Th17 cell responses that became pathogenic rather than protective, with an IL-17/macrophage inflammatory protein-2- (MIP-2-) dependent influx of neutrophils and induction of lung pathology rather than containment of infection [30].

The crossregulation of Th1 and Th17 populations seems to be crucial for protection against MTb to reduce the inflammation-induced damage [31]. It has been shown that IFN-*γ* inhibits the production of IL-17 by CD4^+^ T cells, reducing the survival of neutrophils and the accumulation of these cells in infected lungs contributing to a diminution of the inflammation [32]. These data suggest that IFN-*γ* appears to limit the population IL-17-producing cells.

Despite the important role of IFN-*γ*, IL-12, and IL-17 in the fight against MTb, several studies based on the knockout mice model have shown that also TNF-*α*, granulocyte-macrophage colony-stimulating factor (GM-CSF), IL-1, and IL-6 are crucial components of MTb control growth [33].

In particular, TNF-*α* has been associated with the maintenance of granuloma integrity, and changes in its levels have been correlated with disease susceptibility both in experimental models and in human patients [34–37]. In fact, TNF-*α* acts synergistically with IFN-*γ* to stimulate the production of nitric oxide (NO) by macrophages and influences the expression of chemokines, such as CCL5, CCL9, CXCL10, and CCL2, which induce migration to and maintenance of immune cells in the infection site [38].

IL-1*β* is essential for host resistance to MTb, as shown in the murine model in which IL-1*β* decreases MTb replication activating the innate antimicrobial activity through the recruitment of TNF, upregulation of cell surface TNFR expression, and caspase-3 activation [39]. Moreover, IL-1*β* contributes to the host protection against MTb through the induction of PGE2 synthesis regulating in this way also its own production. In particular, PGE2 decreases type I IFN response [40] which is increased in active TB [41] and it is a down modulator of IL-1 secretion in addition to other cytokines required for effective MTb clearance (i.e., IL-12 and TNF-*α* [42, 43]).

IL-6 has both pro- and anti-inflammatory properties and it is involved in the Th17 and Th22 cell differentiation both important for antimycobacterial activity [44, 31]. It is produced early during mycobacterial infection and is involved in macrophage and cytotoxic T-cell differentiation [45]. Lethal TB has been described in IL-6-deficient mutant mice [46].

**Figure 1:**
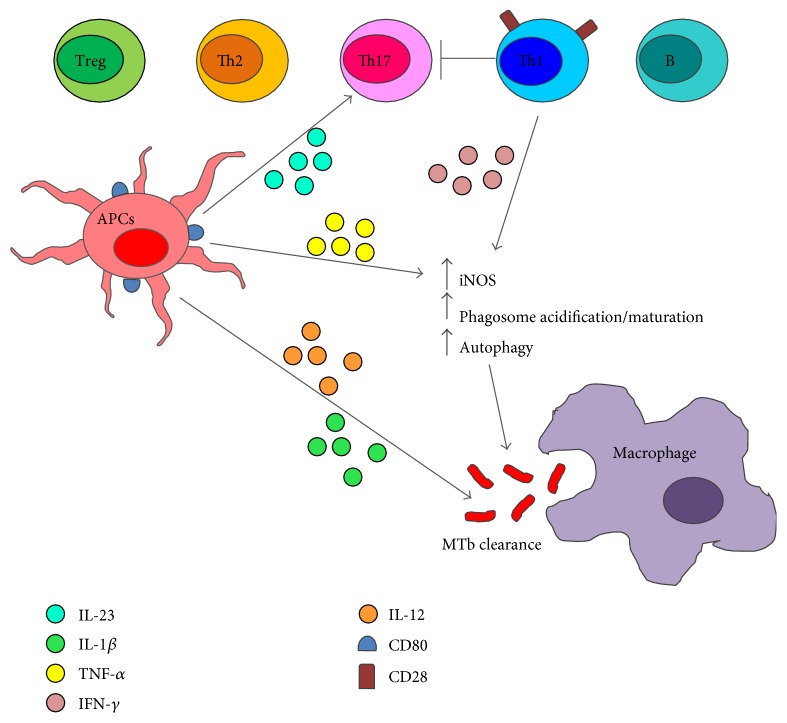
Schematic representation of the immune cells involved in Mycobacterium tuberculosis infection. Distinct types of T helper (Th) cells as Th1, Th2, Th17, and regulatory T cells (Treg) are present at the site of Mycobacterium tuberculosis (MTb) infection. These cells exert their functions mainly through soluble factors. In particular, Th1 cells producing IFN-γ play an essential role in MTb clearance enhancing the macrophage microbicidal mechanisms through the activation of the iNOS pathway and the induction of phagosomes acidification, maturation, and autophagy. Moreover, tumor necrosis factor- (TNF-) *α*, produced by antigen presenting cells (APCs) after MTb stimulation, acts synergically with IFN-*γ* thus contributing to MTb control. APCs produce also interleukin- (IL-) 12 and IL-1*β* that are essential for resistance to MTb. Moreover, IL-23 produced by APC induces the differentiation of Th17 cells producing IL-17, IL-17F, IL-6, and TNF-*α*. Th17 cells are associated with MTb protection; however, when Th17 cell responses became pathogenic rather than protective, Th1 cells are induced to stop these dangerous effects. Finally, the role of Th2, Treg, and B-cell subsets in human disease still remains controversial and needs further elucidations.

As summarized in Figure 1, immune protection against TB depends on several immune components. Here, we analyzed mainly the crossregulation of Th1 and Th17 populations and the production of TNF-*α*, IL-12, IL-1, and IL-6 as important players in TB control.

### 3.3. Review of Methods to Detect LTBI: Sensitivity, Specificity, Confounding Factors, and Limits

From an operational point of view, LTBI is defined as a state of persistent immune response to MTb antigens detected either by the tuberculin skin test (TST) or by IGRA without evidence of clinically manifested tuberculosis. Therefore, LTBI subjects carry an increased risk of progression to TB which is augmented in LTBI individuals with immune impairment as HIV coinfection [47] or therapies with TNF-*α* inhibitors [1, 3, 48, 49] or other immune regulators used for inflammatory diseases and transplantation [50] or compromised immunity due to noncommunicable diseases, such as type 2 diabetes [51, 52].

The TST and IGRA tests are based on immunological sensitization to mycobacterial antigens. TST response is quantified by the skin induration resulting from intradermal injection of purified protein derivative (PPD), a crude mixture of antigens, many of which are shared by MTb, *M. bovis*, *Bacillus Calmette et Guérin* (BCG) and several species of environmental mycobacteria. Blood-based IGRA, including QuantiFERON TB Gold In-Tube (Qiagen; QFT-GIT) and T-SPOT.TB (Oxford Immunotec), measures in vitro IFN-*γ* production upon antigen stimulation of the whole blood by enzyme-linked immunosorbent assay (ELISA) or peripheral blood mononuclear cells (PBMC) by enzyme-linked immunospot (ELISPOT) assay, respectively [53]. The specificity of these assays is due to the stimulation with peptides spanning MTb antigens ESAT-6, CFP-10, and TB7.7 for QFT-GIT that are restricted to a region of the MTb genome deleted from *M. bovis Bacillus Calmette et Guerin* and which is not present in most environmental mycobacteria [54–57].

Advantages of IGRA are due to the fact that they require only a single laboratory test with negative and positive controls and only one visit. Moreover, the in vitro tests may distinguish true negative responses from anergy [53]. Recently, an updated version of the QFT-GIT has been launched [58]. The QuantiFERON TB Plus includes an additional antigen tube to QFT-GIT, which contains peptides that are intended to specifically induce a CD8 T-cell response in addition to the CD4 T-cell response [59] detected with the original QFT-GIT assay [59, 60]. The new CD8-specific peptides have been added to increase the sensitivity of the test because it has been shown that MTb-specific CD8^+^ T cells are mainly associated to active TB [61–66]; that, if detected in LTBI, they are associated with a recent exposure to MTb [67]; and that they decline after anti-TB treatment [64, 68]. The first data on performance of QuantiFERON TB Plus were reported recently [69–72].

A new promising test for LTBI detection is the C-Tb [73] which is a skin test measuring the hypersensitivity to recombinant ESAT-6 and CFP-10 proteins following intradermal administration. The authors claim that it combines the strengths and advantages of TST and IGRA technologies, the ease of use and low cost of TST, and a high-specificity analogous to IGRAs. Another test, based on Rv3615c encoded outside the RD1 region, has been shown to have potential as a new T-cell-based immunodiagnostic [74, 75].

Beside the advantages, TST and IGRAs present limitations. They are characterized by low accuracy in immune-compromised patients and cannot distinguish between LTBI and active TB disease [53]. The latter is a major issue in TB-endemic areas and leads to poor predictive value for development of TB in persons with LTBI [53, 75, 76]. Therefore, it is crucial to find biomarkers that can differentiate between active and quiescent bacterial replication in persons with LTBI or host markers that identify those with LTBI who are at risk of developing active disease [62, 77–84].

### 3.4. Non-Anti-TNF-Targeted Biologics for RA

Table 1 summarized the current licensed biologics for the treatment of inflammatory disorders and their action on cells and cytokines of immune response.

#### 3.4.1. Tocilizumab

TCZ is a recombinant, humanised, monoclonal, anti-IL-6 receptor antibody competing for both the membrane-bound and soluble forms of human IL-6 receptor with inhibition of the binding of IL-6 to its receptors and its proinflammatory activity. The drug, both for intravenous or subcutaneous administration, is currently approved combined or in monotherapy for the treatment of rheumatoid arthritis (RA). The literature search disclosed 30 clinical trials of 15,485 patients with RA with a clinical observation ranging from 14 weeks to 5 years [85–114]. Notably, in 19 studies, LTBI screening procedures and TB reactivation prophylaxis were not included in the protocol as an inclusion criteria [85–104]. Overall, no TB cases were observed, though the 24-week duration of most studies may have led to underestimate the occurrence of active TB. However, also in the long-term trial, active TB cases did not occur [102, 109–114]. Equally, no TB cases were recorded in the only one registry of 302 RA patients treated with TCZ [115].

In a postmarketing Japanese surveillance [116], 4 cases of active TB were observed in 3881 patients. TB occurred after with an interval ranging from 24 days to 4 months after the beginning of TCZ therapy, with an incidence of 0.22/100/year, which is lower than the reported incidence of 15/100/year in Japan [117].

In the real life, 8 patients receiving TCZ and developing active TB were reported [118], but all cases were observed in countries at high TB risk, including Thailand, Spain, South Africa, Peru, Singapore, Brazil, and Mexico.

To summarize, data on a large number of TCZ exposed from clinical trials indicate a very low or absent risk of TB reactivation. Sporadic cases reported from the daily clinical practice occurred in high TB risk countries, thus raising the concern of a primary TB infection rather than LTBI reactivation.

#### 3.4.2. Rituximab

RTX is a chimeric mouse-human monoclonal antibody that selectively depletes the CD20^+^ peripheral B-cell subpopulation via multiple mechanisms, including antibody-dependent cellular toxicity, complement-mediated lysis, and induction of apoptosis [119]. RTX obtained the FDA approval for the treatment of RA in 2006 at the standardized dosage of 2 infusions of 1000 mg each at a 2-week interval with retreatment after 6 months. As expected, due to its B-lymphocytes-targeted action, no cases of active TB were recorded in 9 RCTs of RTX in a total number of 3623 patients with RA [120–128] and in 9 open-label studies in 1191 patients with the same disease [129–137]. An analysis of 3194 RA patients recruited in 8 RCTs and 2 long-term open-label extension trials followed for 9.5 years and receiving up to 17 RTX courses disclosed 2 cases of active pulmonary TB [138]. Data on the demographic and clinical features of these 2 patients, concomitant therapies, additional risk factors for TB, and the country of origin are not available. Previously, in a survey from the Infectious Diseases Society of America, members of the Emerging Infections Network, 3 cases of active TB were reported in patients treated with RTX, but no further description of these patients is available [139]. Recently, a nationwide retrospective cohort study from Taiwan, a country at high TB risk, reported 2 cases of active TB in 763 patients with RA treated with RTX [140]. These 2 patients had a history of previous anti-TNF treatment, and active TB occurred after 8 and 10 years of RTX therapy.

Another case of TB knee arthritis in a 42-year-old woman patient receiving RTX has been published [141]. This patient was not screened for LTBI and she was living in Cambodia, a country at high TB risk.

Confirming the low risk of TB reactivation, no cases of active TB were observed in 1303 RA patients included in the French AIR registry who had received at least 2 RTX courses [142], in 370 patients with different autoimmune diseases in the GRAID registry from Germany [143], in 234 in Greece [144], while 1 TB case was recorded in 2484 RTX-exposed RA patients in the German GERINIS registry [145].

The low or absent risk of TB reactivation associated with RTX administration has been confirmed by two other studies on 56 RA patients at high TB risk [146] and on patients previously treated for active TB [142, 147, 148].

In conclusion, in patients with rheumatic diseases receiving RTX, the TB risk is negligible, and according to the Rituximab Consensus Expert Committee [149], the screening procedures for LTBI before therapy starting seem unnecessary.

#### 3.4.3. Abatacept

ABA is a soluble fully human fusion protein blocking the activation of T cells by binding with costimulatory proteins present on APCs (CD80/86 on APCs and CD28 on T cells) [150]. The drug has been approved for the treatment of RA and it is administered intravenously every 4 weeks at the dose of 10 mg/Kg or subcutaneously at a weekly dose of 125 mg.

Though the reduced expression of CD8/CD28 T cells exerted by ABA may reduce the immune response against TB infection [151], only 1 case of presumptive active TB was registered in 17 trials of ABA administered either intravenously or subcutaneously in 8539 patients with RA [150, 152–167]. The reported TB case was described in the 3-year-extension phase of the AIM study [168]. An ABA-exposed 39-year-old woman was diagnosed as having TB because she was responsive to TB therapy, but bronchial lavage and biopsy were negative. Two more cases of active TB were observed in the 5- and 7-year long-term extension phases of the same trial [169, 170].

Data from real-life practice showed no TB cases in 682 patients included in the ORA French registry [171], and no single case description of TB in patients treated with ABA is reported.

More recently, no TB cases occurred in 231 ABA-exposed patients who were enrolled in a Japanese multicentre registry [172].

To conclude, active TB in ABA-exposed patients is a rare event and, far apart, less frequent than that in the general population, thus suggesting the screening procedures for LTBI as unnecessary.

### 3.5. Non-Anti-TNF-Targeted Biologics for PsA and AS

#### 3.5.1. Ustekinumab

UTK is a fully human monoclonal antibody directed against the p40 subunit common to IL-12 and IL-23, thereby blocking the interaction of IL-12 and IL-23 with their cell surface receptors [173]. Consequently, the inflammatory pathways Th1 IL-12 and Th17 IL-23 dependent, which are strongly implicated in the pathogenesis of psoriasis and PsA, are inhibited [174]. Based upon 1 phase II [175] and 2 large phase III trials [176, 177] enrolling a total of 1073 patients, UTK was approved for the treatment of PsA. Beyond its efficacy, UTK demonstrated an excellent safety profile with respect to overall infections. No cases of active TB were recorded in the 3 trials both in a short-term period and after 2 years of treatment [178]. Moreover, in a subanalysis of 5 trials of UTK in psoriasis and PsA, no cases of active TB developed in 167 patients positive for LTBI [179]. Confirming the absence of TB reactivation risk, data from the Psoriasis Longitudinal Assessment and Registry (PSOLAR), including 3474 patients with psoriasis and PsA receiving UTK, indicate the absence of TB cases over a median follow-up of 1.60 years [180]. Therefore, UTK may ensure an effective and safe treatment in LTBI-positive PsA patients.

#### 3.5.2. Secukinumab

The results of efficacy and safety from 3 controlled trials of PsA [181–183] and 2 trials of AS [184, 185], respectively, enrolling a total number 1045 and 620 patients, led to the approval of SCK, a fully human monoclonal antibody targeting and neutralizing IL-17A, for the treatment of the two rheumatic disorders. No cases of TB reactivation were recorded, and the same resulted from a pooled safety analysis of 10 studies of SCK in psoriasis [186].

SCK has been recently marketed and data from real-life clinical practice are lacking. However, its mechanism of action and the safety results from clinical studies indicate that the drug is safe with respect to TB reactivation and may represent a good therapeutic option in patients with PsA and AS who are at increased risk of TB.

## 4. Discussion

Over time, in developed countries, TB has been characterized by a fluctuating epidemiology with a peak in the 18th and 19th centuries and a progressive reduction of incidence over the 20th century [187]. A resurgence of the disease was recorded after the introduction of anti-TNF-*α*-targeted therapies for the treatment of RA, PsA, and AS. Indeed, TNF-*α* blocking negatively interferes with the TB granuloma formation and maintenance and the growth of MTb, thus facilitating the reactivation of TB [4]. The problem was reduced after the adoption of screening procedures to detect LTBI; however, an increased risk of TB reactivation in anti-TNF-exposed patients is still currently observed [4].

During the last 10 years, new inflammatory pathways sustained by CD20 and CD28 lymphocytes and cytokines other than TNF-*α*, including IL-6, IL-12, IL-23, and IL-17, have been discovered with consequent development of biologics directed against these new targets.

Theoretically, these new targeted therapies, acting on cells of cytokines scarcely or not involved in the immune response against the TB infection, would be safer than anti-TNFs in LTBI-positive patients with RA, PsA, and AS. As expected, in our review, we found reassuring data concerning the risk of TB reactivation associated with TCZ, RTX, and ABA in patients with RA and with UTK and SCK in those with PsA and AS, respectively. Indeed, only sporadic cases of active TB, not exceeding the frequency of the disease in general population, were reported in TCZ-, RTX-, and ABA-exposed patients with RA, and no cases were associated with UTK and SCK in patients with PsA and AS (Table 2). In our opinion, these data greatly influence the management of patients with RA, PsA, and AS requiring biologic therapies. Indeed, LTBI positivity represents an important variable for choosing the first-line biologic therapy. Current recommendations suggest anti-TNFs as the first-line therapy for RA [188, 189], but TB reactivation risk should be taken into account by clinicians, and in the case of LTBI diagnosis, a non-anti-TNF-*α*-targeted biologic would probably represent the best choice in terms of efficacy and safety. Other variables should guide the choice among TCZ, ABA, and RTX, including the need for monotherapy, anticitrullinated protein antibodies positivity, and preferred administration route by the patient [190].

The 2015 updated Group for Research and Assessment of Psoriasis and Psoriatic Arthritis (GRAPPA) recommendations for the treatment of PsA and psoriasis include UTK (SEK was not approved at the time of publication) among the biological therapies and consider several variables driving the choice toward an anti-TNF-*α* as first-line therapy [191]. However, among the considered variables, the authors did not include the possibility of patients with a diagnosis of LTBI. As previously stated in this paper, based on the absence of evidence of an increased TB reactivation risk in patients with PsA receiving UTK or SCK, we suggest to use these non-anti-TNF-targeted biologics as first-line therapy in PsA patients with LTBI.

The same is true for AS treatment. Despite the recent SCK approval, current guidelines recommend anti-TNF as the only choice to treat AS patients with active disease [192, 193] independently on LTBI screening procedures. Nowadays, in our opinion, considering the efficacy and safety of IL-17 inhibition, AS patients with positive LTBI tests should be treated with SCK as first-line biologic.

## 5. Conclusion

The availability of non-anti-TNF-targeted biologics has widened the therapeutic strategies in patients with RA, PsA, and AS, allowing optimization in the biologic choice of function of several clinical variables. Among these, the TB reactivation risk should be assessed in all patients, and in case of positive results, non-anti-TNF-*α*-targeted biologics for RA, UTK, or SCK for PsA, and SCK for AS represent the safest option.

## Figures and Tables

**Table 1:** Immune cells and factors involved in the immunity against tuberculosis. List of some of the biological drugs used in the treatment of rheumatological disorders that inhibit immune paths.

Cytokine	Producing cell type	Role in tuberculosis	Biological drug inhibiting this path	References
IFN-γ	T lymphocytes, NK	(1) Activates iNOS pathway (2) Induces the process of acidification and maturation of phagosomes (3) Induces autophagy (4) Inhibits IL-17 production		[22, 23–26, 32]
TNF-*α*	T lymphocytes, macrophages	(1) Maintain of granuloma integrity (2) Changes in TNF-*α* levels have been correlated with disease susceptibility (3) Acts synergistically with IFN-*γ* to stimulate the production of NO by macrophages (4) Influences the expression of chemokines	Infliximab, etanercept, adalimumab, abatacept	[34–38]
IL-12	Macrophages, dendritic cells	(1) Individuals with mutations in the IL-12/IFN-*γ* axis develop disseminated infection caused by BCG or nontuberculous species of mycobacteria (2) Important for MTb clearance	Ustekinumab	[27, 42, 43]
IL-23	Macrophages, dendritic cells	(1) Mice repeatedly exposed to MTb and BCG developed strong IL-23-induced Th17 cell pathogenic responses	Ustekinumab	[30]
IL-6	T lymphocytes, macrophages	(1) Pro- and anti-inflammatory properties (2) Involved in the Th17 and Th22 cell differentiation (3) Early produced during mycobacterial infection (4) Involved in macrophage and cytotoxic T-cell differentiation (5) IL-6-deficient mice develops lethal TB	Tocilizumab	[31, 44–46]
IL-17	CD4 T cells	(1) Has protective immunity against hyper-virulent MTb strains (2) Removal of IL-17-producing cells enhanced recruitment of Th1 cells to the lung (3) Has pathogenic role of Th17 cells during chronic infection with MTb or BCG in mice	Secukinumab	[28–30]
IL-1*β*	Macrophages	(1) Decreases MTb replication activating the innate antimicrobial activity (2) Induces PGE2 synthesis that leads to a decrease of type I IFN response	Anakinra	

IFN: interferon; TNF: tumor necrosis factor; IL: interleukin; NK: natural killer; iNOS: inducible nitric oxide synthase; NO: nitric oxide; BCG: Bacillus Calmette-Guérin; MTb: Mycobacterium tuberculosis; Th: T helper; PGE2: Prostaglandin E2.

**Table 2:** Non-anti-TNF-targeted biologics: reported TB cases from national registries and postmarketing surveillance.

Biologic	Country; patient N°	TB cases	IR	Expected IR/100/year (WHO)	Reference
Tocilizumab	Japan; 3881	4	0.22	15–100	[116]
	Japan; 302	0	0	15–100	[115]
Rituximab	France; 1303	0	0	10–24	[142]
	Germany; 370	0	0	10–24	[143]
	Germany; 2484	1	0.12	10–24	[145]
	Greece; 234	0	0	10–24	[144]
	Taiwan; 763	2	0.38	15–100	[140]
Abatacept	France; 682	0	0	10–24	[171]
	Japan; 231	0	0	15–100	[172]
Ustekinumab	Worldwide; 3474	0	0	NA	[180]
Secukinumab	Unavailable data	NA	NA	NA	NA

WHO: World Health Organization-estimated incidence of TB, 2016; NA: not applicable.
